# Supplementary material for: Analyzing the relationship between productivity and human communication in an organizational setting
Source: PLoS One. 2021 Jul 14;16(7):e0250301. doi: 10.1371/journal.pone.0250301 (PMC8279403; doi:10.1371/journal.pone.0250301)
Supplement: S1 Appendix — (PDF) [file pone.0250301.s001.pdf]

S1 Appendix. Speech signal features. Various speech features were extracted that carry information about speaker identity, language, accent, various aspects of affect, etc. It is a common method of reducing the dimension of a speech signal, while maintaining the perceptive power of the signal. We denote $x_i(n), n = 1, ..., W_L$ as the sequence of audio samples of the ith frame, where W_L is the length of the frame. A total of 12 types of features were investigated in this study and can be subdivided as follows,

Pitch

Pitch is defined as the rate of periodic vibration of the vocal cords and is also referred to as the fundamental frequency. Detailed analysis of fundamental frequency (f_0) and its harmonics helps in understanding of emotion dependent pitch modulation observed in expressive speech. The basic problem is to extract the f_0 from a sound signal, which is usually the lowest frequency component, or partial, which relates well to most of the other partials. In a periodic waveform, most partials are harmonically related, meaning that the frequency of most of the partials are related to the frequency of the lowest partial by a small whole-number ratio. The frequencies $\omega = \frac{2\pi}{k} f_0$ are referred to as the harmonics of wave, where k is the propagation constant. There are a lot of ways to estimate the pitch distribution, and in our case we calculated the autocorrelation function of the signal to estimate it [1]. The features, related to pitch distribution are described below.

- *Fundamental frequency (f_0) or pitch frequency*
- *Harmonics*: 1-12 harmonics can be used. Changes in harmonics come from the changing shape of the vocal folds during speech.
- *Hamonic ratio*: This is a measure of the harmonic to noise ratio, which provides an indication of the overall periodicity of the signal by quantifying the ratio between the periodic (harmonic) and the non-periodic (noise) part [2].

Temporal features

The time-domain features are

- *Zero crossing rate*: This is a measure of dominant frequency or the number of time domain zero-crossings within the speech frame, and is calculated as the rate of sign changes along each frame of the signal [3]. It is computed as,

$$Z(i) = \frac{1}{2W_L} \sum_{n=1}^{W_L} |sgn[x_i(n)] - sgn[x_i(n-1)]|,$$

  where $sgn(.)$ is the sign function.

- *Energy*: Short-time energy distinguishes voiced speech from unvoiced speech and evaluates the amplitude variation and power of the signal for each frame. It is calculated as,

$$E(i) = \sum_{n=1}^{W_L} |x_i(n)|^2$$

- *Energy entropy*: This can be interpreted as a measure of abrupt changes in the energy level [4], which might correspond to reactions to something in the environment or conversation. We have used 100 milliseconds as a short term window to measure the entropy. It is calculated using the ratio of each sub frame (j) energy and the total energy of the frame.

$$e_j = \frac{E_{subFrame_j}}{E_{Frame_i}}$$

  The energy entropy is given by,

$$H(i) = -\sum_{j=1}^{k} e_j \cdot \log_2(e_j)$$

Spectral features

These features are computed in the frequency-domain and they provide a convenient representation of the distribution of the frequency content of the signal. In order to proceed, let $X_i(k), k = 1, ..., W_{FL}$, be the magnitude of the DFT coefficients of the ith audio frame. The different frequency domain features are,

- *Spectral centroid*: This is a measure of the center of gravity of the spectrum of the signal frame. A higher value of spectral centroid corresponds to a brighter sound. It is calculated as,

$$C_i = \frac{\sum_{k=1}^{W_{FL}} kX_i(k)}{\sum_{k=1}^{W_{FL}} X_i(k)}$$

- *Spectral spread*: This is the second central moment of the spectrum which measures how the spectrum is distributed around its centroid which is commonly associated with the bandwidth of the signal. Individual tonal sounds with isolated peaks result in a low spectral spread, so its variation indicates various forms of affect. It is calculated as,

$$S_i = \frac{\sum_{k=1}^{W_{FL}} (k - C_i)^2 X_i(k)}{\sum_{k=1}^{W_{FL}} X_i(k)}$$

- *Spectral entropy*: Similar to energy entropy, this computes the abrupt changes in the spectrum [5]. We first divide the spectrum of the short-term frame into L sub-bands (bins). The energy E_f of the fth sub-band, $f = 0, ..., L-1$, is then normalized by the total spectral energy, that is, $n_f = \frac{E_f}{\sum_{f=0}^{L-1} E_f}$, $f = 0, ..., L-1$. The entropy of the normalized spectral energy n_f is finally computed according to the equation,

$$H = -\sum_{f=0}^{L-1} n_f \cdot \log_2(n_f)$$

- *Spectral flux*: This measures the spectral change between two successive frames and is calculated as the squared difference between the normalized magnitudes of the spectra of the successive frames [6]. It evaluates the temporal variation in speech. It is computed as,

$$Fl_{(i,i-1)} = \sum_{k=1}^{W_{FL}} (EN_i(k) - EN_{i-1}(k))^2$$

  where, $EN_i(k)$ is the kth normalized DFT coefficient of the ith frame.

- *Spectral roll-off*: It is defined as the frequency below which 90% of the spectral distribution is concentrated [7]. It helps discriminating between voiced and unvoiced part of speech and studying its variation across time can capture different aspects of emotions like stress, anger etc.

Cepstral features

Cepstral features are calculated by taking the inverse fourier transform of the logarithm of the estimated spectrum of the signal. The power cepstrum has been used widely for speech analysis.

- *Mel-frequency cepstrum coefficients (MFCC)*: These are perhaps the most popular features that has been used successfully in speech emotion recognition problems [8, 9]. It is a type of cepstral representation of signal, where frequency bands are distributed according to the mel-scale, which are similar to human auditory system. The coefficients are the discrete cosine transform of the mel-scaled log-power spectrum. We have used the first 13 MFCCs as they are considered to carry enough discriminative information.

We have calculated various statistics of these features over periods of time to study the changes in these features over time.

Network graph features. A finite graph can be represented in matrix forms. An adjacency matrix is a square matrix used to represent a finite graph, in which the elements of the matrix indicate whether pairs of vertices are adjacent or not in the graph. In graph theory, a degree of a node is defined as the number of edges incident on the node. A degree matrix is a diagonal matrix which contains information about the degree of each node. Several topological features that aim to describe the nature of daily interactions can be extracted from these graphs. A total of 11 graph features were investigated in this work.

Basic graph descriptors

- *Number of edges*: The total number of edges or links present in the graph.

- *Number of nodes*: The total number of active nodes present in the graph.

- *Average degree*: It is the number of links per node, and defined as $\frac{2m}{n}$, where n is the number of nodes and m is the number of edges.
- *Number of connected triples*: This routine counts the number of connected triples of nodes. Here it can be defined as a subgraph of three nodes such that there is at least one node among the three which is adjacent to both of the other two nodes.
- *Number of cycles*: This routine calculates the number of independent loops of cycles. It is defined as $m - n + c$, where m is the number of edges, n is the number of nodes and c is the number of connected components.

Graph centrality measures

Centrality refers to the place of nodes in the network, namely how they are connected to all other nodes in a local or global sense. Generally, there are centralities based on the number of links per node, or based on the number of paths that go through a node. The following features are used as the measures of graph centrality.

- *Degrees*: The average number of edges adjacent to a node.
- *Average neighbor degree*: It is a measure of the average degree of adjacent or neighboring nodes for every vertex. In our work we computed took the average of this measure across all nodes.
- *Eigen centrality*: It is the eigenvector corresponding to the largest eigenvalue of the adjacency matrix. The i-th component of this eigenvector gives the centrality score of the i-th node of the network. The average eigen centrality across all nodes was computed for this study.

Laplacian features

These functions concern mostly the spectrum of the adjacency matrix, for which the Laplacian of the graph has to constructed. The Laplacian graph is defined as the difference between the degree and the adjacency matrix. The Laplacian matrix $L_{n\times n}$, is defined as,

$$L = D - A,$$

where, D is the degree matrix and A is the adjacency matrix.

- *Graph spectrum*: It is the list of all the eigenvalues of the Laplacian of the graph. An average over all eigen values was used for this study.
- *Algebraic connectivity*: It is the second smallest eigenvalue of the Laplacian graph.
- *Graph energy*: It is defined as the sum of the absolute values of the real components of the eigenvalues of the graph.

Regression Method. The regression methods allow us to summarize and study relationships between two continuous (quantitative) variables. One variable, denoted x, is regarded as the predictor, explanatory, or independent variable, and the other variable, denoted y, is regarded as the response, outcome, or dependent variable. The three regression methods used in this study are briefly described below,

- **Support vector regression**

  In support vector regression, the input X is first mapped onto a m-dimensional feature space using some fixed (nonlinear) mapping, and then a linear model is

constructed in this feature space. Using mathematical notation, the linear model (in the feature space), $\mathbf{y}$ is given by

$$\mathbf{y} = \sum_{j=1}^{m} w_j g_j(X) + b$$

where $g_j, j = 1, ..., m$ denotes a set of linear/nonlinear transformations, and b is the "bias" term. In this work, we have used a second order polynomial transformation. Often the data are assumed to be zero mean (this can be achieved by preprocessing), so the bias term is dropped. The output variable is estimated my minimizing $||w||^2$ and the quality of estimated is measured by a loss function. In Weka, Alex Smola and Bernhard Scholkopf's sequential minimal optimization algorithm for training a support vector regression model [10, 11] is implemented. In this paper, we implemented a second order polynomial kernel function (g_j), in order to establish the relationship between communication and productivity. The accuracy of the SVR model is evaluated by comparing the predicted result with the actual data.

References

1. Rabiner, Lawrence R.. On the Use of Autocorrelation Analysis for Pitch Detection. *IEEE Transactions on Acoustics, Speech, and Signal Processing* (1977).

2. Murphy, P J. Perturbation-free measurement of the harmonics-to-noise ratio in voice signals using pitch synchronous harmonic analysis. *The Journal of the Acoustical Society of America* (1999).

3. Panagiotakis, Costas and Tziritas, George. A speech/music discriminator based on RMS and zero-crossings. *IEEE Transactions on Multimedia* (2005).

4. Pikrakis, Aggelos and Giannakopoulos, Theodoros and Theodoridis, Sergios. Gunshot detection in audio streams from movies by means of dynamic programming and Bayesian Networks. *ICASSP, IEEE International Conference on Acoustics, Speech and Signal Processing - Proceedings* (2008).

5. Misra, Hemant and Ikbal, Shajith and Sivadas, Sunil and Bourlard, Hervé. Multi-resolution spectral entropy feature for robust ASR. *ICASSP, IEEE International Conference on Acoustics, Speech and Signal Processing - Proceedings* (2005).

6. Sadjadi, Seyed Omid and Hansen, John H.L.. Unsupervised speech activity detection using voicing measures and perceptual spectral flux. *IEEE Signal Processing Letters* (2013).

7. Frigo, Matteo and Johnson, Steven G.. FFTW: An adaptive software architecture for the FFT. *ICASSP, IEEE International Conference on Acoustics, Speech and Signal Processing - Proceedings* (1998).

8. Nakagawa, Seiichi and Wang, Longbiao and Ohtsuka, Shinji. FFTW: Speaker identification and verification by combining MFCC and phase information. *IEEE Transactions on Audio, Speech and Language Processing* (2012).

9. Ganchev, Todor and Ganchev, Todor and Fakotakis, Nikos and Fakotakis, Nikos and Kokkinakis, George and Kokkinakis, George. Comparative evaluation of various MFCC implementations on the speaker verification task. *Proceedings of the SPECOM-2005* (2005).

10. Shevade, S. K. and Keerthi, S. S. and Bhattacharyya, C. and Murthy, K. R K. Improvements to the SMO algorithm for SVM regression. *IEEE Transactions on Neural Networks* **11**, 1188–1193 (2000).

11. Smola, Alex J. and Schölkopf, Bernhard. A tutorial on support vector regression. *Statistics and Computing* **14**, 199–222 (2004).
