# Supplementary material for: Analyzing the relationship between productivity and human communication in an organizational setting
Source: PLoS One. 2021 Jul 14;16(7):e0250301. doi: 10.1371/journal.pone.0250301 (PMC8279403; doi:10.1371/journal.pone.0250301)
Supplement: Attachment — Submitted filename: Response to Reviewers.pdf [file pone.0250301.s002.pdf]

Analyzing the relationship between productivity and human communication in an organizational setting

Arindam Dutta, Elena Steiner, Jeffrey Proulx, Visar Berisha, Daniel W. Bliss, Scott Poole, Steven Corman

Response to reviewers:

We would like to sincerely thank the reviewers for their valuable suggestions. All the suggestions were very helpful, and we have tried our best to work on those suggestions and made necessary changes. The response to all the comments made by the reviewers have been addressed below, which also reflects the changes made in the paper.

Reviewer #1: This paper uses very detailed voice recording data from what I think is a kind of student software consulting organization at a University over the course of almost 3 years to study whether measures of voice communication can predict productivity. The voice data used in the study is very unique in how complete it is and how long a time period it covers and it allows the authors to generate measures of communication between pairs of individuals inside the organization (employees and clients). The authors find that speech and communication network characteristics can predict software programmer productivity.

I do not have a sufficient understanding of voice measures to comment on the validity what seems to be heroic work by the authors to generate useful measures of communication networks and speech characteristics.

While I think a better understanding of how communication relates to productivity in organizations is a very important question, I am not convinced this study advances our understanding of this question. There are several reasons for this, which I separate out below.

----

1. The authors do not provide sufficient information about the organization they are studying, except that the work being performed by employees is software programming. The authors argue that the type of work being performed should affect the relationship between communication and productivity, which I agree with, but so should, for instance, the organizational structure, and the sales or work cycles. From what I can gather on my own, it seems as though the software factory is a student run software consultancy which suggests a very flat organizational structure in which most conversations may be between “equals”. It also suggests that work occurs when there is a project available, and otherwise no work occurs. Without understanding how the organization functions and how often work occurs in the organization, it is difficult to know the extent to which the findings generalize to other organizations.

Moreover, the authors mention that their voice data includes conversations between programmers and between programmers and clients. It seems to me like the type of communication that is desirable differs depending on whether a programmer is speaking with a fellow programmer or with a client. I may have missed a discussion of whether the authors drop the client-facing conversations, but I was surprised this wasn’t emphasized more. Given that the measure of productivity used in the paper does not directly capture how satisfied a client was or how big a contract the programmers got, I don’t think the conversations with clients should be included in the study.

Response: We have added additional details about the organizational structure in the **lines 77-108**. We have provided details about the employees, projects and project handling processes. We acknowledge to

the reviewer that the organization was indeed flat, with the managing engineer and everybody else on an equal level below.

----

2. As the authors point out, the study is not able to determine whether different types of communication patterns cause more or less productivity, but rather whether they can be useful to predict how well the organization is doing (in this case, how productive workers will be). For instance, communication frequency may be a proxy for how excited or engaged workers are in the job or how frustrated they are with the client's demands. If speech and communication network characteristics are easier to observe and measure than other things like employee attitudes, that using them to predict productivity seems like it could be a very useful tool for employers to improve performance by helping them catch potential problems before they become disasters.

However, I do not think the authors did enough to explain how feasible or practical it is for employers to adopt this in practice. I imagine many employees would object to being recorded while working, and it may be hard to retain high quality employees if they were required to record themselves while at work. Given the importance of communication frequency for predicting productivity, partial recordings may not be sufficient. Generating useful data out of the voice recordings would also be expensive timewise. Thus, it is unclear to me what the practical takeaways of the paper are.

Response: We do not intend the method to be applied by other organizations for practical purposes. Our immediate purpose in comparing productivity to detected interaction was to validate our detection method, i.e. to prove that the communication we detected has expected relationships to organizational outcomes. An additional purpose was to support a larger sponsored project, focused on discrepancies between observable and perceived communication. We have made these clarifications and cited a related study on **lines 68-73**.

----

3. I don't think my previous comment would be important if the study improved our understanding of why people communicate the way they do when they are more or less productive. As I mention above, there are many reasons why communication patterns may change at the same time as productivity changes but the authors do not tell us anything about these potential mechanisms. I think the authors could do more with their data in this regard. For instance, perhaps they could get data on when project deadlines are and test whether the relationship between communication out productivity changes when workers are facing more urgency. Similarly, perhaps the authors could get information on when students are working on different types of jobs (e.g. maybe students participated in hack-a-thons at some points that were not contracted for). These types of tests may move us forward in our understanding of how speech and communication patterns differ depending on the circumstances, and how those differences relate to productivity.

Response: We acknowledge the reviewer's comments and agree that such analyses could be illuminating. However, we are using historical data, and it does not contain information about client deadlines. In any case, deadlines were not a prominent feature of SF work because it used Xtreme Programming (XP) as its software development process. XP is an incremental development process and is not focused so much on deadlines as it is development-feedback cycles. There were also no job types that were out of the ordinary (such as hackathons). It could be that different project types have different relationships to productivity. We have added this as a suggestion for future research in **lines 454-457**.

----

4. This is a more minor comment, but I wondered if employees would choose to delete or not turn in recordings of less appropriate discussions, including disagreements. If this is possible, perhaps this might

explain the lack of findings on speech signal features? Some discussion of possible measurement error of these features might be helpful for understanding how much we can take away from the findings.

Response: This could have happened, but it would have been reflected in problems time-aligning recordings for our cross-correlation analyses, and we saw no evidence of it. Also, people involved in SF said that after the first week or so, members tended to forget the recorders. The same has been reported in other studies doing long-term recording of participants. We have mentioned this in **lines 117-121** of our limitations section.

----

5. I am also a bit concerned about mismeasurement of productivity in the lines of code changed may not capture code quality. The authors acknowledge that deletions may or may not improve code quality, but on the converse, additions may or may not improve code quality. At the extreme, if one programmer is particularly frustrated with a co-worker or client, they may write a number of confusing lines that could make it hard for subsequent programmers to successfully edit the code. There are some relatively standard measures of code quality that the authors could have external programmers evaluate the organization's code on (e.g. complexity, coupling).

Response: We acknowledge lines of code is a measure of productivity, but not the only one, and agree this is a limitation. On the other hand, according to software engineering scholars we contacted, measuring software quality is far from straightforward or settled science. To our knowledge, there are not any gold standards, and what methods exist require qualitative analysis of code, which is not practical for the amount of code in the SF repository. The fact that there was a multitude of projects, varying in fundamental concepts, requirements and programming languages (the coding was done in multiple platforms, for example C, C++, Java, Fortran, Labview, etc.), complicates possible quality evaluation even more.

Reviewer #2: I think this is great. I like the analyses to measure communication among this group of employees. The sample size is a bit small for my taste, but overall it's quite sound. I think this could be an important paper that change the way we measure communication in the future.

Response: We thank the reviewer for the encouraging comments.
