# Supplementary material for: Analyzing the relationship between productivity and human communication in an organizational setting
Source: PLoS One. 2021 Jul 14;16(7):e0250301. doi: 10.1371/journal.pone.0250301 (PMC8279403; doi:10.1371/journal.pone.0250301)
Supplement: Attachment — Submitted filename: Response to Reviewers Round2.pdf [file pone.0250301.s003.pdf]

Analyzing the relationship between productivity and human communication in an organizational setting

Arindam Dutta, Elena Steiner, Jeffrey Proulx, Visar Berisha, Daniel W. Bliss,
Scott Poole, Steven Corman

Response to editor:

We reviewed the references and removed the ones which were unused. We have also updated the order of the references based on how they appear in the manuscript.

Response to reviewers:

We would like to sincerely thank the reviewers for their valuable suggestions. All the suggestions were very helpful, and we have tried our best to work on those suggestions and made necessary changes. The response to all the comments made by the reviewers have been addressed below, which also reflects the changes made in the paper.

Reviewer #1: Thank you very much for your response to my comments. I appreciate the additional details on the work setting, and the inclusion of statements about the extent to which employees could have deleted their conversations and about the methods not being intended for adoption by organizations.

However, I don't think you sufficiently addressed my concern about speech patterns and productivity being both affected by some additional variable (I suggested deadlines, but it could also be the different stages of the task as outlined by the authors in the revised draft). Thus, it may look like certain speech patterns contribute to productivity in the data but in reality, it could be that certain job stages drive certain speech patterns and those same job stages generate different amounts of coding. I think this is an important limitation of the current paper, and at a minimum, should be mentioned in the conclusion as such.

Response: Indeed, there is a causal relationship between changes in speech pattern and job stage, that is driving the overall productivity (in terms of amounts of coding) as well. We acknowledge that this study does not capture how various job stages in a project influence the speech pattern and communication volume, resulting in changes in overall productivity. We have mentioned this in lines 457-464 as a limitation of this study.

Reviewer #2: As I said before, I think this is an interesting study that could have important contributions to the field. I think it should be published.

Response: We thank the reviewer for the encouraging comments.
